# Modelling shifts in agroclimate and crop cultivar response under climate change

**DOI:** 10.1002/ece3.782

**Published:** 2013-09-30

**Authors:** Reimund P Rötter, Jukka Höhn, Mirek Trnka, Stefan Fronzek, Timothy R Carter, Helena Kahiluoto

**Affiliations:** 1 Plant Production Research, MTT Agrifood Research FinlandLönnrotinkatu 5, FI-50100, Mikkeli, Finland; 2 Plant Production Research, MTT Agrifood Research FinlandVakolantie 55, FI-03400, Vihti, Finland; 3 Institute of Agrosystems and Bioclimatology, Mendel University in BrnoZemedelska 1, 61300, Brno, Czech Republic; 4 Global Change Research Centre, Academy of Science of the Czech RepublicBelidla 986/4a, 60300, Brno, Czech Republic; 5 Climate Change Programme, Finnish Environment Institute (SYKE)P.O. Box 140, FI-00251, Helsinki, Finland

**Keywords:** Adaptation, agroclimatic indicator, barley, crop simulation model, cultivar response diversity

## Abstract

This paper aims: (i) to identify at national scale areas where crop yield formation is currently most prone to climate-induced stresses, (ii) to evaluate how the severity of these stresses is likely to develop in time and space, and (iii) to appraise and quantify the performance of two strategies for adapting crop cultivation to a wide range of (uncertain) climate change projections. To this end we made use of extensive climate, crop, and soil data, and of two modelling tools: N-AgriCLIM and the WOFOST crop simulation model. N-AgriCLIM was developed for the automatic generation of indicators describing basic agroclimatic conditions and was applied over the whole of Finland. WOFOST was used to simulate detailed crop responses at four representative locations. N-AgriCLIM calculations have been performed nationally for 3829 grid boxes at a 10 × 10 km resolution and for 32 climate scenarios. Ranges of projected shifts in indicator values for heat, drought and other crop-relevant stresses across the scenarios vary widely – so do the spatial patterns of change. Overall, under reference climate the most risk-prone areas for spring cereals are found in south-west Finland, shifting to south-east Finland towards the end of this century. Conditions for grass are likely to improve. WOFOST simulation results suggest that CO_2_ fertilization and adjusted sowing combined can lead to small yield increases of current barley cultivars under most climate scenarios on favourable soils, but not under extreme climate scenarios and poor soils. This information can be valuable for appraising alternative adaptation strategies. It facilitates the identification of regions in which climatic changes might be rapid or otherwise notable for crop production, requiring a more detailed evaluation of adaptation measures. The results also suggest that utilizing the diversity of cultivar responses seems beneficial given the high uncertainty in climate change projections.

## Introduction

Agricultural production is sensitive to variations in weather and climate and can be expected to be influenced markedly by climate change (Rosenzweig and Hillel 1998; Rötter and van de Geijn 1999; Parry et al. 2004; Fischer et al. 2005; Godfray et al. 2010). Global warming is expected to lead to rapid increases in temperature, especially in northerly latitudes (Betts et al. 2011; Ruosteenoja et al. 2011). Future projections of precipitation mainly show increases in northern Europe, which are usually largest in winter (Fronzek et al. 2012), but with considerable variation between climate models (Sloth Madsen et al. 2012). Projected changes in mean climatic conditions have generally been considered beneficial for agriculture in the Nordic region (e.g., Carter et al. 1996). However, recently doubts have been raised whether that also holds true if climatic variability increases markedly and progress in plant breeding and agronomy cannot keep pace ensuring effective adaptation (Rötter et al. 2011a). Eventually, implementation of effective adaptation might also be hindered by too high uncertainties in climate change projections. The current study aims at a detailed national assessment of climate change risks to crop production that, for the first time, systematically combines an agroclimatic indicator approach with crop growth simulation using the same daily input data.

Most studies of climate change impacts on crop yields apply either statistical models (Lobell and Burke 2010) or process-based crop simulation models (Rötter et al. 2011b; White et al. 2011; Osborne et al. 2013). Most process-based models are also capable of simulating, in addition, effects of enhanced CO_2_ concentration and management practices on biomass, seed yields and water use of crops (Rosenzweig and Parry 1994; Nelson et al. 2009; Ewert et al. 2011; Angulo et al. 2013). However, even the more complex process-based crop simulation models cannot take all important interactions between the environment and management (E × M) into account, such as effects of heavy rainfall on harvested yield. Neither do they include all interactions between genotype and environment (G × E) such as yield reduction due to weather-induced pest and/or disease occurrence. On the other hand, crop growth simulation is the only meaningful practical way for analysing the interactions between the many options of combining different crop cultivars with diverse management practices under a wide range of possible new environmental conditions (Semenov and Halford 2009; Rötter et al. 2011a,b; Rosenzweig et al. 2013). Usually, crop-climate models do not cover all important crops and soils in a region. For this reason, agroclimatic indicator approaches are sometimes applied to provide a more comprehensive picture of the agroclimate for larger areas and its shifts under climate change (Harrison and Butterfield 1996; Trnka et al. 2011). Knowledge of the broad-scale agroclimate can also provide a useful basis for upscaling site specific crop simulation results, offering a strong argument for combining the two approaches. Such a combination can provide information on shifts in the suitability and potential for crop production in a region or country under climate change (Carter and Saarikko 1996; Challinor 2011).

This study demonstrates the benefit of combining agroclimatic indicators calculated with gridded weather data for Finland with more detailed crop growth simulations. It covers one of the few regions in Europe where the changing climate is expected to improve overall agroclimatic conditions (Carter et al. 1996; Trnka et al. 2011), but where concerns still remain about the ability to utilize the potential due to specific soil conditions, increased pest and disease risks, the rapid rate of the change and possible increasing climate variability.

**Figure 1:**
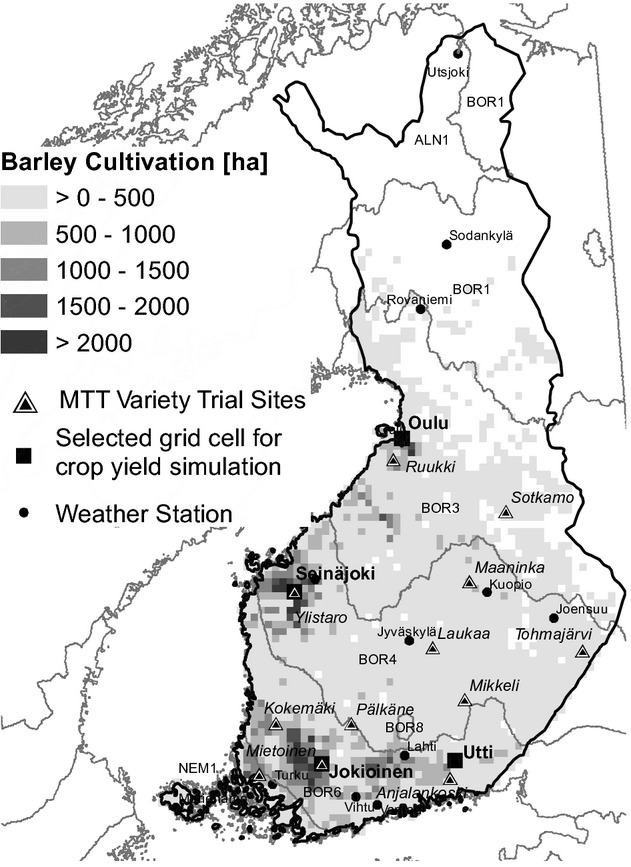
Barley cultivation, weather stations, major MTT official variety trial sites and Environmental Zones (EnZs) for Finland according to Metzger et al. (2005). Triangles indicate locations of MTT official variety trial sites for barley. Filled large squares indicate selected grid used for crop yield simulation in this study (small filled circles indicate long-term weather stations).

The specific objectives of this paper are: (i) to identify areas in Finland where crop yield formation is currently most prone to climate-induced stresses, (ii) to evaluate how the severity of these stresses is likely to develop in time and space under a wide range of future climate projections, and, based on such risk assessment, (iii) to appraise and quantify the performance of two alternative strategies for adapting crop cultivation to uncertain projections of future climate. To exemplify this, we use spring barley (*Hordeum vulgare* L.) as test crop and daily weather data for the baseline period (1971–2000) and a wide range of projected futures (32 climate scenarios) up to year 2100, at a spatial resolution of 10 × 10 km for the entire country. Barley (see, photo) is the most widely grown field crop in Finland - its cultivation area is shown in Fig. 1. Results of the study are expected to provide fundamental knowledge for target-oriented plant breeding and agronomic advancements designed to enhance the resilience of agricultural systems under a changing climate in Finland.

## Materials and Methods

### Set-up of the study

To assess shifts in the agroclimatic suitability of major crops and in the yield potential of current cultivars of spring barley (as a key crop) in Finland, we applied a combination of two impact assessment methods that are usually applied separately. First, the AgriCLIM software to calculate agroclimatic indicators (Trnka et al. 2011) was extended to include indicators relevant for higher latitudes in a version called N-AgriCLIM. A description of how these indicators were selected is given in the Data S1. The tool was applied to assess shifts in agroclimatic suitability for cultivating crop- and grassland, and identify areas most prone to climatic risks under a wide range of climate change scenarios. Second, the process-based dynamic crop simulation model WOFOST (version 7.1; van Diepen et al. 1989; Boogaard et al. 1998) was applied to quantify impacts of climate change on yields for different currently available barley cultivars and for a large ensemble of climate change scenarios.

Both N-AgriCLIM and WOFOST were run with the same daily weather data on a 10 × 10 km^2^ grid basis for the period 1971–2100. While N-AgriCLIM was run for the whole of Finland, WOFOST simulations were conducted only for selected grid cells (see, Fig. 1), and with soil data for representative soil types. Crop data applied in N-AgriCLIM were based on characteristics of the popular barley cultivar Scarlett, while the more comprehensive crop data required for crop modelling were extracted and processed from MTT official variety trial databases (e.g., Kangas et al. 2006).

N-AgriCLIM, developed from AgriCLIM (Trnka et al. 2011) that had been used to calculate agroclimatic indicators selected on the basis of a previous Europe-wide study, was applied to undertake subsequent statistical analysis of the relationships between yield of spring barley cultivars and weather variables in Finland (Hakala et al. 2012) (see, Table S1). Out of that analysis a final set of 10 agroclimatic indicators was selected, which were deemed most relevant for Finnish agriculture, capturing conditions that have the most pronounced influence on growth and yield formation of major Finnish crops. These comprise: (i) the sum of effective global radiation (Egr), (ii) number of effective growing days (Egd), (iii) date of the last frost (LastFrost), (iv) relative sowing date (DelayS) and (v) proportion of suitable days for sowing in spring (Sowing), (vi) number of days with water deficit during the period from April to June (DryAJ) and (vii) June to August (DryJA), (viii) total precipitation during the period from 3 to 7 weeks after sowing (RainAS), (ix) number of days with maximum temperature of 28°C or higher 1 week before to 3 weeks after heading (StressE) and (x) temperature sum accumulation rate during period between anthesis and physiological maturity, that is, grain filling (TempHRAvg); definitions are provided in Table 1. As discussed for example, by Trnka et al. (2011), indicators i-v are much more important to grass and perennial crops than they are for annual field crops such as cereals.

**Table 1:** The 10 selected agro-climatic indicators generated by N-AgriCLIM (Trnka et al. 2011) (as presented in Fig. 6)

Agroclimatic indicator	Indicator name (units)	Definition	Symbol
Potential biomass and crop development	Sum of effective global radiation (MJ m-2 season -1)	Sum of global radiation of days with daily mean temperature >5°C, daily minimum temperature >0°C, ETa^*^/ETr ratio >0.4 and no snow cover	Egr
Time period suitable for crop growth	Sum of effective growing days (days)	Number of days with daily mean temperature >5°C, daily minimum temperature >0°C, ETa^*^/ETr ratio >0.4 and no snow cover	Egd
Low temperature limitations	Date of the last frost (date from January 1st)	Last occurrence of a daily minimum temperature of < −0.1°C in the given season before June 30th	LastFrost
Sowing conditions that will affect the growing season	Delayed sowing (day)^1^	Day of the year when 10-day moving average of daily mean temperature exceeds threshold temperature of 8°C expressed as deviation from May 1st	DelayS
	Proportion of suitable days for sowing in time window April 26^th^ through May 20th (late spring)	All days with soil-water content in the top 0.1 m between 10% and 70% of the maximum soil water-holding capacity (SWC), mean daily temperature on the given day and on the preceding day >5°C, without snow cover and with precipitation on the given day <= 1 mm and precipitation on the preceding day <= 5 mm	Sowing
Water deficit during growing season that may result in drought	Number of days with water deficits from April to June (days)	All days within the given period with ETa/ETr of <0.4	DryAJ
	Number of days with water deficits from June to August (days)	All days within the given period with ETa/ETr of <0.4	DryJA
	Rain after sowing (mm)	Sum of rain 3–7 weeks after sowing	RainAS
Potential grain number formation and yield potential determination^2^	Very high temperature stress (days)	Number of days with maximum temperature of 28°C or higher 1 week before to 2 weeks after heading	StressE
	Mean daily temperature sum accumulation rate at grain filling	Rate of Tsum above 0°C accumulation (per day) from heading to yellow ripeness	TempHRAvg

ETa and ETr stand for actual evapotranspiration and reference evapotranspiration respectively calculated according to FAO methods (Allen et al. 1998) considering spring barley as a cover crop.

1 Carter and Saarikko (1996).

2 Hakala et al. (2012).

Agroclimatic indicators were calculated for each cell of the 10 × 10 km^2^ gridded database and mapped. Details on N-AgriCLIM, for example, on determining relevant crop phenological stages or water deficits, are described in the methods section of the (Data S1).

### Multiple regression analyses of agroclimatic indicators on yield

Observed yields from barley trials (between 1971–2009) conducted at three locations (Jokioinen, Ylistaro and Ruukki) (Fig. 1) were collected to perform multiple regression analyses on the relationship between yield and the various agroclimatic indicators used in this study. The degree of fitness of the models differs by location (Table S2) (see also, Results section).

### WOFOST crop simulation model

The crop model WOFOST (WOrld FOod Studies, version 7.1, Boogaard et al. 1998), developed in the framework of an interdisciplinary study on world food production potentials for annual crops, was applied. Its principal components, process formulations, and various applications have been described by van Diepen et al. (1989) and van Ittersum et al. (2003). The model provides a dynamic description of phenological development, CO_2_ assimilation, respiration, partitioning of assimilates to various plant organs, growth and yield formation and (evapo-) transpiration of a crop from emergence until maturity (at a daily time step), on the basis of crop genetic characteristics, environmental conditions and management practices (G × E × M interactions). WOFOST had been calibrated and applied for different Finnish and European barley cultivars (Rötter et al. 2011b, 2012) with daily weather, soil and crop data established for Finnish conditions. Yield simulations were performed for selected grid cells that are close (within 10 km distance) to long-term variety trial sites, and represent the most important barley cultivation areas and the major environmental zones (Metzger et al. 2005) relevant for agriculture (Fig. 1).

### Input data: crop, soil and current weather

First we grouped available modern barley cultivars (released after 1985) as grown by Finnish farmers, into three groups, depending on their maturity class, naming them after widely known individual cultivars: Annabell (late maturing), Kustaa (medium), and Kunnari (early). As a starting point, we used crop parameters for spring barley based on multi-locational field experiments for individual cultivars (Rötter et al. 2011b). MTT's official variety trial data (Kangas et al. 2006) were used to adjust phenology-related crop parameters for the medium (Kustaa) and early (Kunnari) maturing groups (see, Table S6). Furthermore, we modified crop parameters to account for the enhanced net photosynthesis and increased water use efficiency (Rötter and van de Geijn 1999) due to three different levels of elevated atmospheric CO_2._ The values of those parameters affected by the level of atmospheric CO_2_ concentration (see, Table S7) differed slightly from a previous study (Rötter et al. 2011a) due to small differences in the CO_2_ concentrations considered.

Atmospheric CO_2_ for the next decade is expected to increase at rates between 2 and 4 ppmv per annum (Anderson and Bows 2008). This implies that by 2025 (midpoint of 2011–2040) we may reach levels of approximately 420–450 ppmv; for 2055 this would be 480–570 ppmv and 540–690 ppmv by 2085. Accordingly, we adjusted crop parameters for concentrations of 435, 525 and 615 ppmv, respectively, using established procedures (Table S7). Soil and topographic data comprised field data for volumetric soil moisture (SM) content at saturation (SM0 or total pore space), at field capacity (SMFC) and at wilting point (SMW) and a transmission zone permeability parameter (SOPE) for the root zone. Run-off was assumed to be absent. Plant available soil moisture (PASM) content is calculated as the actual amount available at field capacity minus the content at wilting point (SMFC-SMW); data were derived for a clay loam and a silty sand soil with PASM values of 0.18 and 0.22 (cm^3^/cm^3^), respectively.

Daily weather data interpolated from stations to a regular 10 × 10 km grid were obtained from the Finnish Meteorological Institute covering the period 1971–2009 for the following variables: minimum and maximum (Tmax) near-surface temperature, global radiation, precipitation and vapour pressure (Venäläinen et al. 2005; updated). As mean daily wind speed was not available from this data set, we bi-linearly interpolated the daily mean values of 10-m wind speed from two re-analysis data products provided by the European Centre for Medium-Range Weather Forecasts (ECMWF) from their original, coarser spatial resolution to the 10 × 10 km grid. The re-analysis data sets ERA-interim (Dee et al. 2011) and ERA-40 (Uppala et al. 2005) give a high temporal, but relative low spatial resolution with 0.75° grid cell size for ERA-interim and 2.5° for ERA-40. ERA-40 was used for the 1971–1978 and ERA-interim for 1979–2010. The resulting time series of interpolated values provides a general spatial pattern of differences in wind speed for example between coastal and inner-land areas and compared relatively well with data from selected stations.

### Climate scenarios

**Figure 2:**
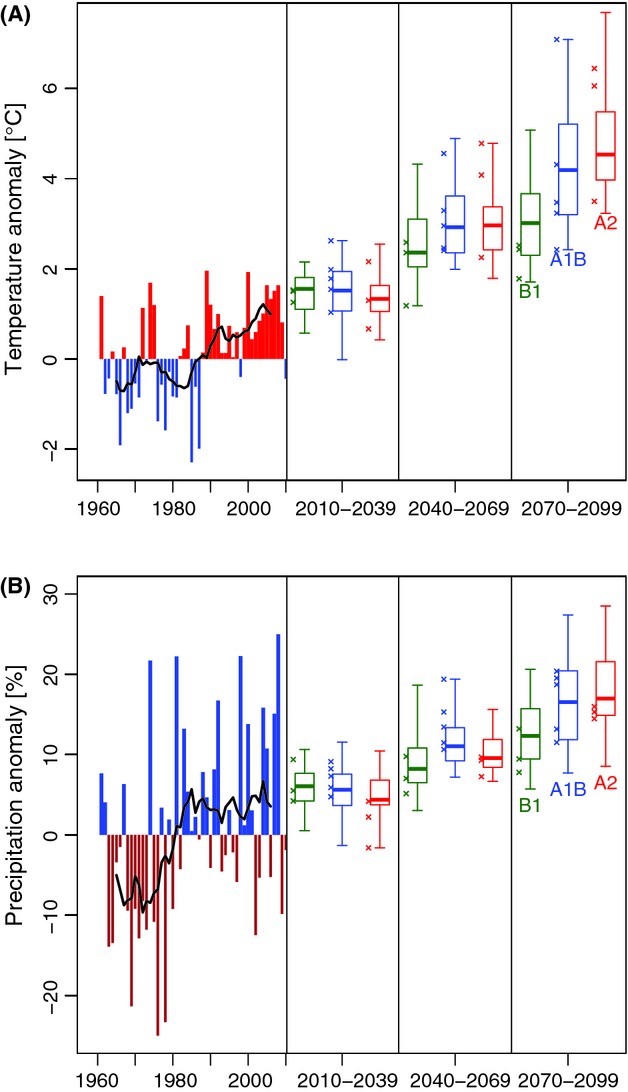
(A–B) Observed anomalies in average annual (A) air temperature and (B) precipitation and projected changes during the 21st century for Finland for SRES (Special Report on Emissions Scenarios (Nakicenovic et al. 2000) scenarios B1, A1B and A2 simulated with 11 GCMs selected for this study (stars, see Table S4) and for a larger ensemble of 24 GCMs (boxplots).

Data from the CMIP3 archive (Meehl et al. 2007) was downloaded representing monthly mean values of output from General Circulation Models (GCMs). Simulations from four experiments were used, one with greenhouse gas concentrations as observed for the 20th century and three forcing scenarios for the 21st century, SRES A2 (high emission), SRES A1B (moderate) and SRES B1 (low) (Nakicenovic et al. 2000). Data have been downloaded for all GCM-SRES combinations for which the required set of variables (Table S4) was available. This resulted in an ensemble of 36 simulations, 13 forced by SRES B1, 14 by A1B and 9 by A2 (Table S4), out of which a sub-set of 11 scenarios has been selected spanning the range of uncertainty. Figure 2 shows for entire Finland historical anomalies as well as projected changes in temperature and precipitation. Climate change scenarios were calculated in three steps:

1. Calculation of monthly long-term mean changes for three future periods, 2011–2040, 2041–2070 and 2071–2100, relative to the baseline period 1971–2000 on the original GCM grid for temperature, precipitation (as relative change), wind speed (calculated from its zonal and meridional components), vapour pressure change and global radiation. Change in vapour pressure was estimated using an approximate relation to sea level air pressure and specific humidity (Mitchell et al. 2004, p. 9).
2. Bi-linear interpolation to the 10 × 10 km grid of the observed data.
3. For each 10 km grid cell, linear interpolation of monthly changes to daily estimates for leap- and non-leap years. The daily deltas were then added to the observed time-series 1971–2000 for each grid cell for the three future periods. The resulting scenarios are therefore reproducing the observed interannual and daily variability and only changes in mean conditions are considered.

### Adaptation measures

Apart from examining the performance of different current barley cultivar groups (characterized in Table S6) under changed climatic conditions, we also took into account the CO_2_ fertilization effect (Table S7). Moreover, we adjusted sowing dates based on established temperature criteria (Carter and Saarikko 1996), but with corrections for differences observed between calculated optimal and actually observed sowing of farmers, who in the majority show a more conservative behaviour in adjusting sowing (on average 1 week later) than would be possible in response to temperature conditions alone.

## Results

### Agroclimatic indicators and their projected shifts by 2025, 2055 and 2085

Multiple regression analyses on the relationship between yield and the various agroclimatic indicators showed that results are location-specific. The highest coefficient of variation (adjusted *R*^2^) is observed for Ruukki trial site, explaining up to 46.6% of the variation of grain yields, whereas the equivalent value is only 33.3% for Jokioinen and 23.8% for Ylistaro. The importance of predictor variables also varies from site to site (see, Tables S2 and S3).

**Figure 3:**
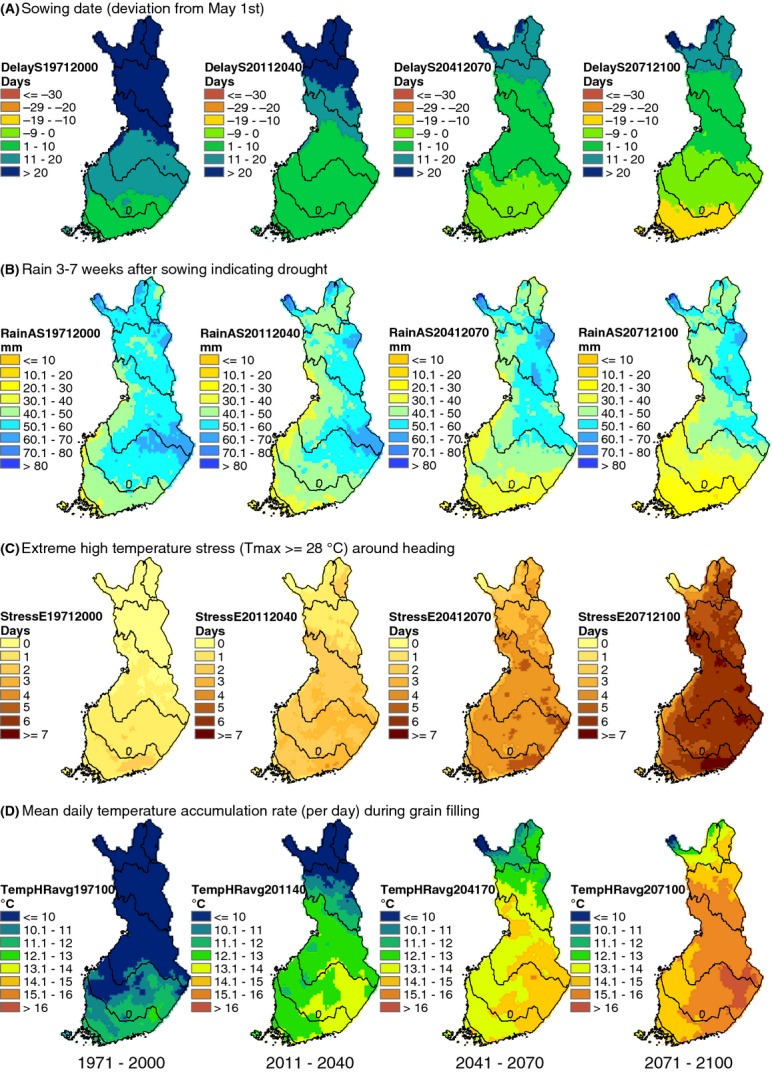
Projected changes for (A) sowing date (DelayS, deviations relative to fixed date 1st May) and three agroclimatic indicators: (B) early drought stress (RainAS), (C) specific heat stress (StressE), and (D) mean daily temperature accumulation rate at grain filling (TempHRAvg, higher value signals higher likelihood for yield reduction), for climate scenario IPSL-CM4/A2. The legend caption contains the abbreviation of the indicator (see Table 1) and the observed time period (e.g., DelayS1140 = sowing date expressed as deviation from May 1st for the time period 2011–2040).

**Figure 4:**
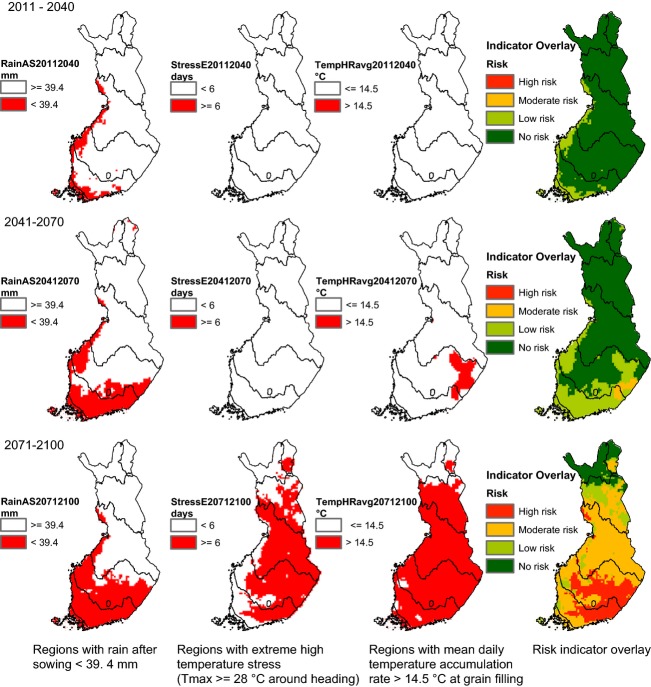
Spatial patterns of the most risk prone areas for each of these indicators using pre-determined thresholds, as well as, the overlay of all three risk factors – IPSL-CM 4/A2 - for each of the three future time slices (2011–2040, 2041–2070 and 2071–2100).

**Figure 5:**
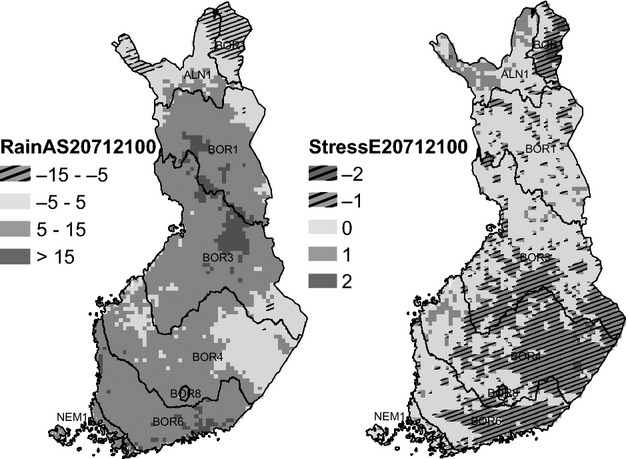
Differences in precipitation sum 3–7 weeks after sowing (RainAS) in mm, and very high temperature stress (StressE) in days, between MIROC3.2(medres)/A1B and IPSL-CM4/A2 scenario. The two difference maps show the deviation values for MIROC3.2 relative to IPSL-CM4.

**Figure 6:**
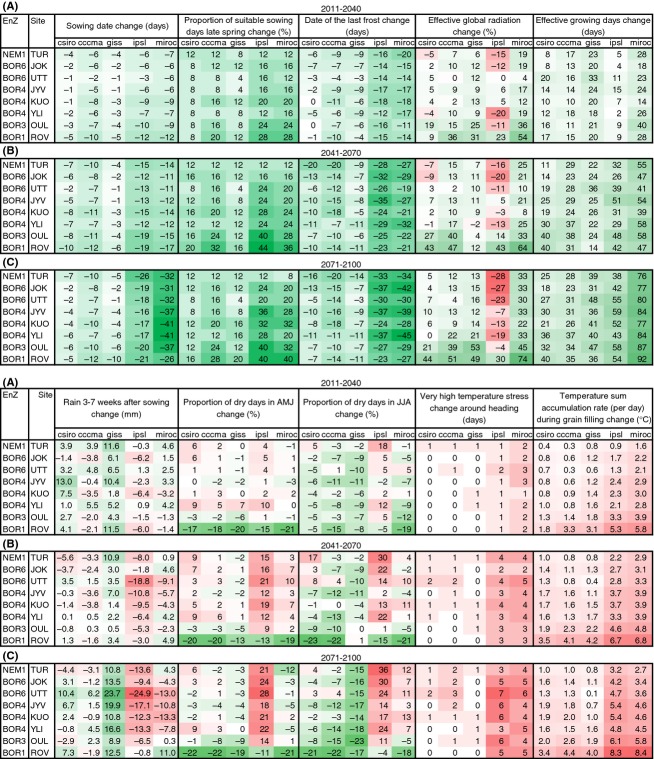
Changes in the median values of selected (10) agroclimatic indicators relative to the 1971–2000 reference period for (A) 2011–2040, (B) 2041–2070, (C) 2071–2100. Estimates based on five GCMs, that is, CSIRO-MK3.5/B1 (csiro), CCCMA-CGCM3.1(T63)/A1B (cccma), GISS-ER/B1 (giss), IPSL-CM4/ A2 (ipsl) and MIROC3.2(medres)/A1B. The key to site abbreviations given is as follows: TUR = Turku; JOK = Jokioinen; UTT = Utti, JYV = Jyväskylä, KUO = Kuopio, YLI = Ylistaro, OUL = Oulu, ROV = Rovaniemi (see, Fig. 1 for their location).

From the 10 agroclimatic indicators (results presented in Fig. 6), three were selected for presentation in form of maps (Figs. 3, 4 and 5): (i) RainAS, (ii) StressE, and (iii) TempHRAvg. The selection was based on a literature review (e.g., Carter and Saarikko 1996; Hakala et al. 2012) and multiple regression analysis for spring barley. Since barley is a good indicator for many other determinate (spring) cereal crops (Rötter and van de Geijn 1999), we can assume that under recent past and present-day climate, the three indicators mentioned above, can be considered most important: They indicate known phenomena such as early season drought (Rötter et al. 2012), heat temperature stress during most sensitive phase around flowering (e.g., Porter and Gawith 1999), and high temperatures during grainfilling period hastening maturity and, thereby, reducing yield potential (e.g., Hakala et al. 2012). We hypothesized that these risks are likely to be further exacerbated under future climatic conditions. In order to provide climate risk information for other crops, including perennials such as grass, we also present results for the seven other indicators (Fig. 6).

Figure 3 shows the projected changes in the three indicators for one climate scenario (IPSL-CM4/ A2 – warm and dry, see Table 2). Results for a contrasting scenario (MIROC3.2(medres)/A1B – warm and wet) have been generated (shown in Fig. S2). Figure 4 illustrates the spatial patterns of the most risk prone areas for each of these indicators for scenario IPSL-CM4/A2 using pre-determined thresholds (Hakala et al. 2012), as well as, their overlay for three future time slices (2011–2040, 2041–70 and 2071–2100, respectively) (Fig. S3 shows this for scenario MIROC3.2(medres)/ A1B). Figure 5 uses two indicators (RainAS and StressE) to illustrate indicator discrepancies resulting from differences in the climate projections of IPSL-CM4/A2 and MIROC3.2(medres)/A1B.

**Table 2:** Projected changes in mean temperature (T-change) and total precipitation (P-change) relative to the baseline climate, 1971–2000, averaged over the whole of Finland from selected climate model simulations for the: (A) summer half-year (March–August) and (B) winter half-year (September–February). Climate models are detailed in Table S4

Summer Climate model simulation	2011–2040		2011–2040		2041–2070	
			
	T-change (°C)	P-change (%)	T-change (°C)	P-change (%)	T-change (°C)	P-change (%)
(A)						
BCCR-BCM2.0/A2	0.9	−0.7	3.0	6.4	4.4	12.6
CCCMA-CGCM3.1/A1B	1.4	3.7	2.2	9.8	2.3	11.4
CSIRO-Mk3.5/B1	1.1	2.4	2.1	3.2	1.8	4.6
GISS-ER/B1	1.2	8.8	1.4	6.7	1.6	20.3
IPSL-CM4/A2	2.2	1.6	4.3	1.1	5.6	0.2
MIROC3. 2(medres)/A1B	2.5	7.2	4.1	10.4	6.4	16.2

Winter Climate model simulation	2011–2040		2041–2070		2011–2040	
			
	T-change (°C)	P-change (%)	T-change (°C)	P-change (%)	T-change (°C)	P-change (%)
(B)						
BCCR-BCM2.0/A2	1.7	−2.8	4.9	6.8	7.4	16.7
CCCMA-CGCM3.1/A1B	1.8	5.3	2.6	11.1	2.5	10.9
CSIRO-Mk3.5/B1	2.1	8.2	3.2	14.4	3.4	14.1
GISS-ER/B1	1.7	12.0	2.8	13.3	2.8	17.1
IPSL-CM4/A2	2.2	6.1	5.4	18.7	7.2	30.2
MIROC3.2(medres)/A1B	2.8	8.3	5.0	13.5	7.5	21.3

Figure 4 shows for scenario IPSL-CM4/A2, period 2011-40, that the most risk prone areas are found along the west and south coast, mainly due to early drought stress falling below the critical value (threshold 39.4 mm). Towards the middle of the century (2041–2070), risk-prone areas expand inland from the west and south, whilst in some smaller areas of south-eastern Finland both early drought and reduced yield potential risk combine, rendering these areas (near Utti, see Fig. 1) the most risk prone in this scenario. By the end of the century (2071–2100), higher risk areas are widespread, covering more than 70% of the country due to exceedance of thresholds for both specific heat stress and reduced yield potential in most areas, whilst areas where all three risk factors exceed the threshold are found mainly in south-eastern Finland. The picture differs distinctly for MIROC3.2(medres)/ A1B (Fig. S3).

Figure 6 (coloured tables with a design modified from Trnka et al. 2011) considers a set of 10 agroclimatic indicators, which have varied relevance across a range of crops including barley. Results are shown for a sample of five climate change scenarios (out of 32) that span the range of climate scenario realizations, thus revealing the uncertainty range in impact projections. Results for the median changes of 10 agroclimatic indicators are given for three time slices (a,b,c) five climate scenarios, and eight locations. The table illustrates considerable differences in indicator values for the different climate scenarios, and also shows large differences between locations. Overall, results suggest that conditions for perennial crops like grass are generally likely to become more favourable (greener shading, as shown especially for indicators 1–5) except for extreme scenario IPSL-CM4/A2. On the other hand, for annual field crops like spring cereals, oilseeds and root crops, conditions tend to deteriorate (redder shading, as shown for indicators 6–10). For spring-sown annual field crops, however, the picture varies more and whether conditions become more or less favourable depends a lot on the climate scenario. The secular variability of the indicator “early drought stress” is shown for the central co-ordinates of four different grid cells in Fig. S4 – for three different climate change scenarios up to the end of the century (30 year time slices 2011–2040, 2041–2070 and 2071–2100, respectively).

### Simulated crop cultivar responses to changes in climate, atmospheric CO_2_ and sowing dates

**Figure 7:**
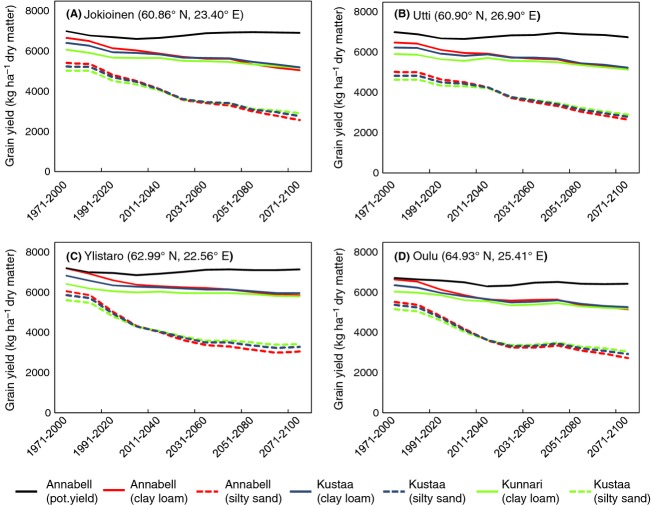
Simulated water limited grain yields (coloured lines) for three cultivar groups representing maturity classes late, medium and early (named Annabell (= late) Kustaa (= medium) and Kunnari (= early), respectively) and potential grain yield (for Annabell only) presented as 30-year moving averages under reference climate and scenario IPSL-CM4/A2 at (A) Jokioinen, (B) Utti, (C) Ylistaro, (D) Oulu for a clay loam and silty sand soil. The *x*-axis indicates the 30-year periods (1971–2000 till 2071–2100).

Drawing from the 11 selected climate scenarios (Table S4), we focused initially on an analysis of a “worst-case” scenario, which projects the lowest precipitation with high warming during summer (March–August) by mid century - IPSL-CM4/A2 (Fig. S1). Simulation results with the WOFOST model for this scenario are illustrated for four grid cells (Fig. 7A-D), representing climatic conditions of the four main concentration areas of barley cultivation (Fig 1.). Results, presented for three current barley cultivar groups assuming potential production (i.e., no limitations of nutrients or water), and for two soil types under water-limited (rainfed) production, indicate some common features, but also distinct differences among the four locations. A common characteristic is the relatively minor variation in simulated potential yield level (range 6.4–7.2 t/ha) over the entire simulation period, 1971–2100.

One effect of future warming is a hastening of phenological development, shortened growth cycle and a reduction of biomass and grain yield. Counteracting this are the positive effects of CO_2_ fertilization and earlier sowing on yield formation, but for potential production these are not sufficient to compensate for development-related losses at all growth stages and locations. Only grid cell “Ylistaro” shows a slight increase in potential yields in the second half of the century compared to the baseline. In contrast, changes in yields attainable under rainfed conditions (attainable yield) show more heterogeneity of response, with the main variation in yield decline attributable to soil type, though location also has a minor contribution to this variation.

For both soil types, clay loam and silty sand, the gap between potential yield and attainable yield widens with time at all four locations. For clay loam, that gap is smallest for Ylistaro, where it is negligible under baseline climate with cultivar Annabell, but with a clear differentiation among cultivars. At the end of the century, there is a gap of about 1 t/ha but hardly any difference in yield responses among cultivars. At the same location, there is also quite a small yield gap for silty sand during the baseline period 1971–2000. However, this follows a rapid yield decline during first half of this century, also showing some differences among cultivars at the end of the century. The simulated yield pattern over time found for Ylistaro most resembles that found at grid cell “Oulu”, though potential yields at Oulu are somewhat lower. For the Jokioinen and Utti grid cells, located further south, the decline of attainable yield is more linear. The yield decline and gap to potential yield are slightly larger at Jokioinen than at Utti, whilst differences in cultivar responses on the clay loam disappear with time. Generally, yield gaps between simulated potential and attainable yields grow from 1 t/ha (Ylistaro, clay loam) and 2.3 t/ha (Utti, silty sand) under baseline climate, up to 4.2 t/ha, or about 45% of potential yield (Jokioinen and Ylistaro, silty sand) at the end of the century.

For one location we considered a wide diversity of (eleven) climate change scenarios (out of 32) in simulating the response of current barley cultivars to changes climate and atmospheric CO_2_ during 2071–2100. For simplification, we assumed an atmospheric CO_2_ concentration of 615 ppmv for all climate scenarios (for details, see Table S7). To examine how the three different cultivar groups respond, we chose a favourable clay loam and single grid cell (Utti) located in the area most prone to high temperature and drought risk (see Fig. 4).

**Figure 8:**
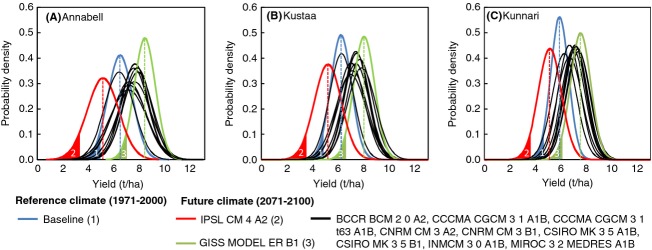
Probability density functions (PDFs) of simulated water limited grain yields for three cultivar groups (from late to early, Annabell, Kustaa and Kunnari) for a clay loam soil under reference climate (1971–2000) and alternative future climatic conditions (2071–2100) at Utti represented by 11 different climate scenarios (for details, see Table S4). PDFs for Baseline (1) and the two most contrasting future climates (2 and 3) are marked specifically. All PDFs assume normal distribution. Kolmogorov-Smirnov test for normality, performed on yield distributions based on crop simulation output for each year presented for each time period and climate scenario, confirmed this assumption. For this test, K-S (NORMAL) function in SPSS statistical software package (version 17.0) was applied.

Figure 8 illustrates (for the clay soil at Utti) how differences in climate change impacts on barley yields were greater between climate scenarios than between cultivar groups. For the late maturing group (Annabell), average yields for the worst case climate scenario (i.e., IPSL-CM4/A2) are 1.2 t/ha lower than for the baseline climate (6.4 t/ha), but they are more than 1.8 t/ha higher for the best case climate scenario (i.e., GISS-ER/B1) (Table 2).

Eight out of the other nine climate scenarios result in higher average yields and yield variability. For the medium maturing (Kustaa) and early maturing (Kunnari) cultivars, yield reductions for IPSL-CM4/A2 are less pronounced, while yield increases under GISS-ER/B1 are of the same order of magnitude (nearly 2 t/ha) as for Annabell. For these two cultivar groups, as for Annabell, most of the climate scenarios also result in average yield increases and higher variability.

### Limits to adaptation with current barley cultivars and agronomic adjustments

Finally, and for the same cultivar groups, we analysed the yield effect of adjusting sowing date as an adaptation measure under two contrasting climate scenarios and for two different soils at one location (Utti) up to the end of the century (see Table 3). Under the high-end scenario (IPSL-CM4/A2) for the near-term period (2011–2040), the different sowings have little effect on yields (Table 3a). There are few yield differences among cultivars and adjusted sowing leads to slightly higher yields on the favourable clay loam, but negligible changes on the less favourable sandy soil. Under mid-century conditions (2041–2070), adjustment of sowing leads to clearer positive yield responses on the clay soil, whereas yields are reduced on the sandy soil. Differences in yield response among cultivars increase over time and reach maximum values at the end of the century, with early cultivars benefitting most from adjusted sowing on the clay loam, while on the sandy soil yields of late cultivars register their lowest values with adjusted (earlier) sowing. In practice this means that on the well-drained silty sands (unlike clay loam), early cultivars increasingly escape early summer drought while late cultivars are affected by terminal drought. Under the moderate (cool and wet) climate scenario GISS-ER/B1, the effects of adjusted sowing are all positive, irrespective of cultivar group and soil type. The yield gains due to adjusted sowing increase over time – and benefits are slightly higher on clay loam than on sandy soil.

**Table 3:** Simulated barley yields with adjusted sowing relative to yields simulated for sowing dates under reference climate for three cultivar groups (Annabell, Kustaa and Kunnari) under two future climates, (A) IPSL-CM4/A2 and (B) GISS-ER/B1 at Utti for a clay loam (CL) and silty sand (SS) soil

		Sowing date (Day of the year)		Yield change (%)	
			
Time period	Cultivar	Reference	Adjusted	CL	SS
(A)					
2011–2040	Annabell	135	130	2.3	0.0
	Kustaa	135	130	1.7	−0.6
	Kunnari	135	130	2.2	0.1
2041–2070	Annabell	135	120	3.4	−8.5
	Kustaa	135	120	4.7	−6.5
	Kunnari	135	120	5.9	−2.6
2071–2100	Annabell	135	115	5.0	−9.4
	Kustaa	135	115	8.1	−6.1
	Kunnari	135	115	11.0	−1.9
(B)					
2011–2040	Annabell	135	132	1.1	0.9
	Kustaa	135	132	1.4	1.4
	Kunnari	135	132	1.5	1.7
2041–2070	Annabell	135	122	3.0	1.6
	Kustaa	135	122	4.0	2.8
	Kunnari	135	122	3.9	2.6
2071–2100	Annabell	135	117	4.3	3.0
	Kustaa	135	117	5.4	4.0
	Kunnari	135	117	5.9	4.4

Overall, the results indicate that for many of the climate scenarios studied we can expect moderate yield increases and slight increases in yield variability for spring cereals on favourable soils towards the end of the century. Moreover, under most scenarios studied, current barley cultivars and adjusted sowing would suffice as adaptation measure. However, there are also some scenarios that would lead to reduced yields - even when the CO_2_ fertilization effect is taken into account. Furthermore, no changes in inter-annual or daily climatic variability were considered in these simulations, which should also be expected to affect average yields and yield variability.

## Discussion

This paper presents a unique high resolution data set at national level for Finland. In combination with crop simulation, it provides an opportunity to examine the implications for crop yield of limited and planned adaptation under a wide range of climate scenarios. The development of our approach has been motivated by a Europe-wide study on agroclimatic conditions for Europe by Trnka et al. (2011). Among the conclusions arising from that study were the suggestions that in order to provide enhanced information for agricultural adaptation: (i) comparable research in future should consider a wider range of climate scenarios, and (ii) regional (sub-national) scale studies using high resolution data would be needed on climate change-induced shifts of agroclimatic indicators. Moreover, both Carter and Saarikko (1996), in early work in Finland, and more recently Challinor (2011) have argued that both agroclimatic indicator and crop simulation approaches have an important role to play in assessing climate change impacts on food production. This finally led us to apply the combined indicator and simulation approach using gridded daily meteorological data.

### Limitations of the study

In spite of its merits, the study has several limitations. First, we only applied the relatively simple delta change approach for down-scaling output from GCMs to generate climate scenario data for impact analysis. Consideration of one more approach, such as using data from bias corrected Regional Climate Models (RCMs) (Rummukainen 2010) that include changes in climate variability, would have made assessment of uncertainties more complete. Secondly, we only applied one crop model for impact analysis, while an increasing number of authors is proposing use of ensemble crop modeling approaches (see, e.g., Rötter et al. 2012; Graux et al. 2013). In this study we applied WOFOST, which is the crop model that has been most extensively applied with data available from modern Finnish crop cultivar trials (Rötter et al. 2011a). As suggested in a number of earlier studies for Europe (e.g., Trnka et al. 2011) and Finland (Rötter et al. 2011a), potential impacts of climate change tend to be stronger (more negative), if increased climatic variability with a higher frequency of extreme weather events are assumed. For that reason, we analysed whether the aggregation of daily weather data to 10 × 10 km^2^ grids levels out extreme values. When analysing this for daily maximum temperature, minimum temperature, precipitation and solar radiation, we found only minimal effects of aggregation (results not shown). However, we cannot rule out that impacts of extreme weather events (especially short-term heat and drought stress) were underestimated in the crop simulations, since WOFOST like most other crop models, is not yet fit for adequately capturing and quantifying impacts of extreme events on crop growth and yield (Rötter et al. 2011b). Future work is planned in which the study data will be applied using a multi-model approach to evaluate uncertainties attributable to imperfect impact modeling. Thirdly, there is of course a wider range of modern barley cultivars than those presented in this study, though in fact the current three groups already span approximately the central 70% (approximately from the 15 to 85th percentile) of variation in phenological development rates of available barley cultivars. Finally, in terms of quantified yield impacts, it is probable that the yield outcomes would have been less positive if temperature and precipitation variability were to increase. In such cases (see, Rötter et al. 2011a; Reyer et al. 2012), considerable plant breeding efforts might be required to restore baseline climatic yield potential – or conceivably more fundamental changes might be implied, such as new cropping systems and other farm structural changes (Mandryk et al. 2012).

### Limits to adaptation

It was shown, that under a wide range of climate scenarios, which are conventionally considered to be equally plausible (although the 2011–2040 low emission pathway already appears to be unrealistic in light of the observed unabated rise in recent emissions – Betts et al. 2011), it is not possible to optimize adaptation. The uncertainties in climate change projections make it inevitable that more flexibility and diversity should be introduced into the response measures (Rötter and van de Geijn 1999; Elmquist et al. 2003; Himanen et al. 2013). One proposed adaptation strategy is to apply, to the extent possible, so-called “no-regret measures” (de Bruin et al. 2009), especially those that can serve both adaptation and mitigation objectives (e.g., more diversified crop rotations – Smith and Olesen 2010). Potential benefits from planned adaptation measures, such as breeding for more multi-stress resistance (e.g., drought, heat and pest resistant crop cultivars) require advanced methods and careful *ex ante* analysis by plant breeders (preferably jointly with crop modellers – see, Semenov and Halford 2009). The current approach facilitates, and can easily be expanded to investigate such planned future adaptation measures, including those resulting from advanced breeding methods (Mayer et al. 2012).

### Advances on existing impact assessment approaches

In the study by Trnka et al. (2011) it has been shown that the number of effective growing days under present climate in the Boreal zone (Metzger et al. 2005) is quite low. While the thermal growing season is projected to increase considerably (by 40–50 days) towards the end of the century (Ruosteenoja et al. 2011), it is also projected that early summer drought will be maintained while rainfall during late summer, autumn and winter will very likely increase considerably (Ylhäisi et al. 2010), potentially worsening both harvest and sowing conditions. While Trnka et al. (2011) were able to show a good differentiation of agroclimate across Europe, local conditions can of course be very diverse (Peltonen-Sainio et al. 2009; Olesen et al. 2011). To adapt agricultural systems to a changing climate such diversity has to be represented adequately. Thus, to inform the most appropriate adjustments in management practices, crops and crop rotations, requires local data on climate-induced risks at a high spatial and temporal resolution. The advantage of Trnka's state-of-the-art indicator approach as compared to some earlier work was that the assessment of climate change effects is based on daily weather, and that point analyses are linked to environmental zones (here: Metzger et al. 2005) to support scaling up results to regional level. However, a disadvantage that has also been typical for most earlier impact studies (e.g., Rosenzweig and Parry 1994) is the relatively small number of weather stations (84) in proportion to the large spatial extent of areas analysed. Both the Rosenzweig and Parry (1994) and Trnka et al. (2011) studies tapped only a small fraction of the available weather station data. On the other hand, if the station network is sufficiently dense to allow for daily weather data to be interpolated to a relatively fine resolution grid, as in this study, computation of indicators across the grid can provide a much better representation of the spatial variation in agroclimatic potential. Of course, a precondition for such an advance is that robust interpolation techniques have been applied that ensure both high quality of the gridded weather data set as well as quantified error estimates.

If there are many agroclimatic indicators, as in this study and in Trnka's analysis, it can sometimes be helpful for decision-makers to reduce this complexity by simplifying the information. One approach is to create composite indicators that reflect the most important stresses, such as drought, heat or frost (see, e.g., Donatelli et al. 2012; Teixeira et al. 2013). However, there are few examples of such composites being applied successfully (e.g., Baettig et al. 2007), as agro-ecological conditions are usually too diverse to rely on a single or few parameters. In addition to a literature review, we also performed extensive multiple regression analysis (see, Data S1) to analyse whether combinations of indicators could be identified that could be related individually and collectively to crop production risks. For spring cereals we found that between four and six indicators might be sufficient, but that these would differ from the primary indicators explaining climate-related variation in yields of another crop (e.g., ley grass). There have also been earlier attempts to link agroclimatic indicator mapping approaches with crop growth simulation (e.g., Donatelli et al. 2012), but in those studies only potential impacts and risks to crop production were simulated, while adaptation was not considered.

Our novel approach overcomes earlier shortcomings by combining three main components: a tool for calculating diverse agroclimatic indicators (N-AgriClim), nationwide, GIS-based high resolution mapping of the spatio-temporal dynamics of the most important risks to crop cultivation, and grid-based crop simulation modeling to assess limits of current adaptation strategies. Combined with enhanced observational and experimental data, improved sampling and regionalization methods, and ensemble crop and economic modeling approaches, this approach offers considerable promise to become an important contributor to regional climate change impact assessment for the agricultural sector. As such, it can provide valuable information to policy makers on potential impacts and for making decisions on adaptation strategies for agriculture.

## Supporting Information

**Data S1.** Methods, Results and References

**Methods**

**Table S1.** Agroclimatic indicators generated by N-AgriCLIM.

**Table S2.** Goodness-of-fit of the models for predicting barley yields from agroclimatic indicators and order of variables' entry (using stepwise selection) presented for each trial site.

**Table S3.** Effects of the tested agroclimatic indicators on barley yields at Jokioinen, Ylistaro and Ruukki.

**Table S4.** List of General Circulation Model (GCM) simulations downloaded from the CMIP3 archive (*Meehl* et al. 2007) for three SRES emission scenarios (B1, A1B, A2) (Nakicenovic et al. 2000) for which all variables required to construct scenario data for crop modeling were available.

**Table S5.** Observed and scenario variables used for crop modeling (cf. Wolf et al., 2012).

**Table S6.** Thermal requirements [°C day] for three modern barley cultivar groups from emergence to flowering (TSUM1), and from flowering to physiological maturity (TSUM2); assuming a common base temperature (TBASE) of 0°C - and indication of differences in other crop parameters.

**Table S7.** Changes of crop parameter set (uniform for all spring barley cultivar groups) for different CO_2_ levels: Specific leaf area (SLA), maximum CO_2_ assimilation rate (AMAX occurring over indicated development stage (DVS)) and correction factor for potential evapotranspiration (CFET) under reference climate (350 ppmv) and enhanced (435, 525 and 615 ppmv) atmospheric CO_2_ concentration (with % changes in relation to current level).

**Results**

**Figure S1.** Projected changes in mean temperature and precipitation during March-August relative to the baseline climate (1971–2000) presented for the time periods 2011–2040, 2041–2070 and 2071–2100 for selected locations (Turku (1), Jokioinen (2), Utti (3), Ylistaro (4), Oulu (5), Rovaniemi (6)) representing the environmental zones most relevant for agricultural production in Finland (see Fig. 1).

**Figure S2.** Projected changes for (A) sowing date (deviations relative to fixed date 1st May) and three agroclimatic indicators: (B) early drought stress, (C) specific heat stress, and (D) yield potential reduction risk, for climate scenario 2 (warm and wet), combining SRES emissions scenario A1B with MIROC3.2 (medres) (see Table S4).

**Figure S3.** Spatial patterns of the most risk prone areas for each of these indicators using pre-determined thresholds, as well as, the overlay of all three risk factors – MIROC3.2(medres)/A1B - for each the three future time slices (2011–2040), (2041–2070) and (2071–2100).

**Figure S4.** Early drought stress (Rain sum 3–7 weeks after sowing) presented as 10-year moving average under current (1971–2009) and projected future climate conditions (2011–2040, 2041–2070, 2071–2100) applying delta change method and preserving the variability of the reference climate (1971–2000) for grid cells (A) Jokioinen, (B) Utti, (C) Ylistaro, (D) Oulu, representing the environmental zones most relevant for agricultural production in Finland (see Fig. 1) Climate change projections based on three GCMs × SRES combinations: GISS-ER/B1, CCCMA-CGCM3.1 (T63)/A1B and IPSL-CM4/A2 (see, Table S4).

**References** Additional references for Data S1
