# Modeling shifts in agroclimate and crop cultivar response under climate change

**DOI:** 10.1002/ece3.890

**Published:** 2013-11-13

**Authors:** Reimund P Rötter, Jukka Höhn, Mirek Trnka, Stefan Fronzek, Timothy R Carter, Helena Kahiluoto

Ecology and Evolution 2013; 3(12): 4197–4214.

DOI: 10.1002/782

The authors of the above paper would like to replace Table 2 with this corrected table below:

**Table 2:** Projected changes in mean temperature (*T*-change) and total precipitation (*P*-change) relative to the baseline climate, 1971–2000, averaged over the whole of Finland from selected climate model simulations for the (a) summer half-year (March–August) and (b) winter half-year (September–February). Climate models are detailed in Table S4

	2011–2040		2041–2070		2071–2100	
			
Climate model simulation	*T*-change (°C)	*P*-change (%)	*T*-change (°C)	*P*-change (%)	*T*-change (°C)	*P*-change (%)
(a) Summer						
BCCR-BCM2.0/A2	0.9	−0.7	3.0	6.4	4.4	12.6
CCCMA-CGCM3.1/A1B	1.4	3.7	2.2	9.8	2.3	11.4
CSIRO-Mk3.5/B1	1.1	2.4	2.1	3.2	1.8	4.6
GISS-ER/B1	1.2	8.8	1.4	6.7	1.6	20.3
IPSL-CM4/A2	2.2	1.6	4.3	1.1	5.6	0.2
MIROC3. 2(medres)/A1B	2.5	7.2	4.1	10.4	6.4	16.2
(b) Winter						
BCCR-BCM2.0/A2	1.7	−2.8	4.9	6.8	7.4	16.7
CCCMA-CGCM3.1/A1B	1.8	5.3	2.6	11.1	2.5	10.9
CSIRO-Mk3.5/B1	2.1	8.2	3.2	14.4	3.4	14.1
GISS-ER/B1	1.7	12.0	2.8	13.3	2.8	17.1
IPSL-CM4/A2	2.2	6.1	5.4	18.7	7.2	30.2
MIROC3.2(medres)/A1B	2.8	8.3	5.0	13.5	7.5	21.3

The time slices in the heading of this table are corrected to be in the sequence 2011–2040, 2041–2070 and 2071–2100.

We would also like to replace the Supporting Information Data S1 with this updated file:

Additional Supporting Information may be found in the online version of this article

**Data S1.** Methods, Results, and References

