# Supplementary material for: Modelling shifts in agroclimate and crop cultivar response under climate change
Source: Ecol Evol. 2013 Sep 30;3(12):4197–214. doi: 10.1002/ece3.782 (PMC3853564; doi:10.1002/ece3.782)
Supplement: Supplementary file 1 [file ece30003-4197-SD1.docx]

**SUPPORTING INFORMATION (SI)**

**Modelling shifts in agroclimate and crop cultivar response under climate change**

R.P. Rötter^1*^, J. Höhn^2^, M. Trnka^3,4^, S. Fronzek^5^, T.R.Carter^5^, H. Kahiluoto^1^

1 Plant Production Research, MTT Agrifood Research Finland, Lönnrotinkatu 5, FI-50100 Mikkeli, Finland

2 Plant Production Research, MTT Agrifood Research Finland, Vakolantie 55, FI-03400 Vihti, Finland

3 Institute of Agrosystems and Bioclimatology, Mendel University in Brno, Zemedelska 1, 61300 Brno, Czech Republic

4 Global Change Research Centre, Academy of Science of the Czech Republic, Belidla 986/4a, 60300 Brno, Czech Republic

5 Climate Change Programme, Finnish Environment Institute (SYKE), P.O. Box 140, FI-00251 Helsinki, Finland

*corresponding author; email: reimund.rotter@mtt.fi

**TABLE OF CONTENTS**

1 Supporting Information Methods

2 Supporting Information Results

3 Additional References for SI

1. **Methods**
   1. Expert system for calculating agroclimatic indices for Nordic conditions (N-AgriCLIM)

*Determination of relevant phenologcial phases*

Several indices including temperature sum accumulation rate, temperature stress and rain after sowing were calculated for different phenological stages of crop development. In order to determine the phenological crop development, growing degree days (GDDs) were used, with GDD sum thresholds defining growth stages. In this current study the spring barley cultivar Scarlett was used as reference crop with following GDDs requirements (base temperature 0^o^ C) for the most important phenological stages after sowing in day degrees (^o^dd): Emergence (80), begin of heading (730), begin of anthesis (780) and maturity (1380). The earliest possible sowing date is set when the 10-day moving average of daily mean temperature exceeds the threshold of 8° C (Carter and Saarikko, 1996; Olesen *et* *al.*, 2012).

*Determination of water deficit*

Effective crop growth is dependent on appropriate temperatures and adequate soil water available for evapotranspiration. Crop growth is considered not to be significantly limited by water if the ratio of daily reference (ETr) and actual (ETa) evapotranspiration exceeds 0.4-0.5 (FAO, 1979; Fisher *et al.*, 2002; Eliasson *et al*., 2007). In this study the lower threshold (0.4) was used to limit eventual overestimation of water shortage. Water deficit parameters were used to evaluate the effective growing season and effective sum of global radiation and to determine the number of days with limited water availability during the period from April to June and from June to August.

All indices were calculated using the software package N-AgriCLIM, which has been originally developed in the framework of a study on agroclimatic conditions in Europe under climate change (Trnka *et al.*, 2011). The original AgriCLIM was extended for the purpose of the project “Enhancing the Adaptive Capacity of the Finnish Agrifood System (Adacapa) through the introduction of additional indices (in Table S1 named WinterL, DelayS, RainBS, RainAS, RainD, RainG, RainR, RainAM, RainS, StressH, StressE, TempBH, TempHR, TempHRAvg ) to better capture agroclimatic aspects (constraints) of high latitudes. N-AgriCLIM uses daily weather data including maximum and minimum temperature, precipitation, global radiation, air humidity (water vapour pressure) and wind speed to calculate a wide range of agroclimatic indices. The full list of indicators generated is given in Table S1.

**Table S1:** Agroclimatic indicators generated by N-AgriCLIM

| **Agroclimatic indicator** | **Indicator name (units)** | **Description** | **Symbol** |
| --- | --- | --- | --- |
| Potential biomass and crop  development | Sum of effective global radiation  (MJm-2 season -1) | Sum of global radiation of days with daily mean temperature > 5°C, daily minimum temperature > 0 °C, ETa*/ETr ratio > 0.4 and no snow cover | Egr ^1)^ |
| Time period suitable for  crop growth | Sum of effective growing days (days) | Number of days with daily mean temperature > 5°C, daily minimum temperature > 0 °C, ETa*/ETr ratio > 0.4 and no snow cover | Egd ^1)^ |
| Low temperature  limitations | Date of the last frost (date from January 1st) | Last occurrence of a daily minimum temperature of < -0.1 °C in the given season before June 30^th^ | LastFrost ^1)^ |
|  | Winter length (°C) | Sum of freezing temperatures | WinterL |
| Sowing conditions that  will affect the growing  season | Delayed sowing (day) | Day of the year when 10-day moving average of daily mean temperature exceeds threshold temperature of 8 °C expressed as deviation from May 1^st^. | DelayS ^3)^ |
|  | Proportion of suitable days for sowing for time window April 26^th^ through May 20th (late spring) ^1)^ | All days with soil-water content in the top 0.1m between 10% and 70% of the maximum soil water-holding capacity (SWC), mean daily temperature on the given day and on the preceding day > 5 °C, without snow cover and with precipitation on the given day <= 1mm and precipitation on the preceding day <= 5mm | Sowing ^1)^ |
|  | Rain for 1 month before sowing (mm) | Sum of rain 4 weeks before sowing | RainBS ^2)^ |
| Water deficit during growing season that may result in drought | Number of days with water deficits from April to June (days) ^1)^ | All days within the given period with ETa/ETr of < 0.4 | DryAJ ^1)^ |
|  | Number of days with water deficits from June to August (days) ^1)^ | All days within the given period with ETa/ETr of < 0.4 | DryJA ^1)^ |
|  | Rain after sowing (mm) | Sum of rain 3-7 weeks after sowing | RainAS ^2)^ |
|  | Number of rainy days during growing period (days) | Number of days during growing period (sowing date +100 days) | RainD |
|  | Precipitation amount during growing period (mm) | Sum of rain from sowing date to sowing date +100 days | RainG |
|  | Precipitation amount between 7 weeks after sowing and sowing date +100 days (mm) |  | RainR |
|  | Precipitation in April + May (mm) |  | RainAM |
|  | Precipitation in Sept. (mm) |  | RainS |
| Potential grain number formation and yield potential determination | High temperature stress (days) | Number of days with maximum temperature of 25 °C or higher 1 week before to 2 weeks after heading | StressH ^2)^ |
|  | Very high temperature stress (days) | Number of days with maximum temperature of 28 °C or higher 1 week before to 2 weeks after heading | StressE ^2)^ |
|  | Rate of temperature sum (Tsum) accumulation before heading (°C) | (Tsum accumulation rate from 14 days before heading to heading | TempBH ^2)^ |
|  | Rate of Tsum accumulation at grain filling (°C) | Tsum accumulation rate from heading to yellow ripeness | TempHR ^2)^ |
|  | Mean daily temperature sum accumulation rate at grain filling | Rate of Tsum above 0°C accumulation (per day) from heading to yellow ripeness | TempHRAvg ^2)^ |

Note: ETa and ETr stand for actual evapotranspiration and reference evapotranspiration respectively calculated according to FAO method (Allen *et al*., 1998) considering spring barley as a cover crop.

^1)^ Trnka *et al*., 2011

^2)^ Hakala *et al*., 2012

^3)^ Carter and Saarikko, 1996

- 1. Multiple regression analysis

A stepwise regression analysis was performed for each trial site entering the variables into the model one at a time in an order determined by the strength of their correlation with the criterion variable. If adding the variable contributes to the predictive power of the model then the variable is retained and another predictor is considered. Otherwise, if it no longer contributes significantly (P > 0.1) the variable is excluded from the model. This method ensures that the smallest possible set of predictor variables is included in the model.

**Table S2:** Goodness-of-fit of the models for predicting barley yields from agroclimatic indicators and order of variables’ entry (using stepwise selection) presented for each trial site.

| Trial Site | Modell | Variables | R | R^2^ | Adjusted R^2^ | Standard error of  estimate |
| --- | --- | --- | --- | --- | --- | --- |
| Jokioinen | 1 | DelayS | 0.454 | 0.206 | 0.205 |  |
|  | 2 | Egd | 0.522 | 0.272 | 0.272 | 1284.3 |
|  | 3 | RainAS | 0.542 | 0.294 | 0.293 | 1265.4 |
|  | 4 | RainR | 0.548 | 0.300 | 0.299 | 1260.0 |
|  | 5 | TempHRAvg | 0.556 | 0.309 | 0.308 | 1252.1 |
|  | 6 | StressE | 0.577 | 0.333 | 0.332 | 1230.2 |
|  | 7 | LastFrost | 0.578 | 0.334 | 0.332 | 1229.5 |
|  | 8 | RainBS | 0.579 | 0.335 | 0.333 | 1229.0 |
| Ylistaro | 1 | RainAS | 0.294 | 0.086 | 0.086 | 1277.3 |
|  | 2 | DelayS | 0.334 | 0.112 | 0.111 | 1259.8 |
|  | 3 | RainR | 0.387 | 0.150 | 0.148 | 1233.1 |
|  | 4 | LastFrost | 0.454 | 0.206 | 0.205 | 1191.4 |
|  | 5 | RainBS | 0.476 | 0.226 | 0.224 | 1176.6 |
|  | 6 | StressE | 0.484 | 0.234 | 0.232 | 1170.9 |
|  | 7 | DryAJ | 0.490 | 0.240 | 0.237 | 1166.9 |
|  | 8 | TempHRAvg | 0.410 | 0.241 | 0.238 | 1166.1 |
| Ruukki | 1 | TempHRAvg | 0.502 | 0.252 | 0.251 | 1246.0 |
|  | 2 | StressE | 0.565 | 0.319 | 0.317 | 1189.4 |
|  | 3 | DelayS | 0.595 | 0.354 | 0.352 | 1159.2 |
|  | 4 | RainBS | 0.630 | 0.397 | 0.395 | 1119.9 |
|  | 5 | RainAS | 0.649 | 0.421 | 0.418 | 1098.3 |
|  | 6 | DryAJ | 0.671 | 0.450 | 0.446 | 1071.5 |
|  | 7 | RainR | 0.675 | 0.455 | 0.451 | 1066.3 |
|  | 8 | Egd | 0.682 | 0.465 | 0.461 | 1057.4 |
|  | 9 | LastFrost | 0.687 | 0.472 | 0.466 | 1051.6 |

**Table S3:** Effects of the tested agroclimatic indicators on barley yields at Jokioinen, Ylistaro and Ruukki. B=estimated yield effect (kg/ha) per parameter unit, S.E.=standard error, Beta= standardised regression coefficient, Sig. = statistical significance of yield response to the indicator

| Trial Site | Variables | B | S.E. | Beta | Sig. |
| --- | --- | --- | --- | --- | --- |
| Jokioinen | (Constant) | 2665.7 | 586.5 |  | 0.000 |
|  | DelayS | -67.3 | 6.1 | -0.26 | 0.000 |
|  | Egd | 25.0 | 1.5 | 0.39 | 0.000 |
|  | RainAS | -10.4 | 1.0 | -0.22 | 0.000 |
|  | RainR | -5.3 | 0.9 | -0.11 | 0.000 |
|  | TempHRAvg | 246.2 | 27.8 | 0.25 | 0.000 |
|  | StressE | -188.3 | 20.1 | -0.21 | 0.000 |
|  | LastFrost | -5.7 | 2.9 | -0.04 | 0.053 |
|  | RainBS | 3.5 | 2.1 | 0.04 | 0.097 |
| Ylistaro | (Constant) | 10553.1 | 612.0 |  | 0.000 |
|  | DelayS | -50.0 | 6.1 | -0.20 | 0.000 |
|  | RainAS | 16.6 | 1.5 | 0.25 | 0.000 |
|  | RainR | -10.3 | 0.7 | -0.37 | 0.000 |
|  | TempHRAvg | 47.2 | 24.4 | 0.06 | 0.053 |
|  | StressE | -108.3 | 19.6 | -0.14 | 0.000 |
|  | LastFrost | -26.1 | 2.3 | -0.28 | 0.000 |
|  | RainBS | -15.0 | 2.0 | -0.17 | 0.000 |
|  | DryAJ | -10.4 | 3.0 | -0.08 | 0.000 |
| Ruukki | (Constant) | 1924.2 | 727.0 |  | 0.008 |
|  | DelayS | -82.8 | 9.0 | -0.29 | 0.000 |
|  | Egd | 12.1 | 2.5 | 0.26 | 0.000 |
|  | RainAS | 10.4 | 1.7 | 0.20 | 0.000 |
|  | RainR | -8.7 | 1.4 | -0.25 | 0.000 |
|  | TempHRAvg | 382.3 | 24.0 | 0.46 | 0.000 |
|  | StressE | -129.8 | 25.4 | -0.15 | 0.000 |
|  | LastFrost | -15.5 | 4.6 | -0.10 | 0.001 |
|  | RainBS | 25.2 | 3.0 | 0.28 | 0.000 |
|  | DryAJ | 30.5 | 3.9 | 0.28 | 0.000 |

- 1. Application of Geographic Information Systems (GIS) for mapping data surfaces

The whole set of agroclimatic indicators was calculated for each grid cell of the 10 x 10 km weather database for all years between 1971 and 2100 in order to determine mean and median indicator values for each time slice under observation (1971-2000, 2011-2040, 2041-2070 and 2071-2100). Those values were finally joined with the original 10 x 10 km grid database to generate maps visualizing the spatial and temporal variation of agroclimatic conditions using ArcGIS software package.

Three agroclimatic indicators including rain 3-7 weeks after sowing (RainAS), very high temperature (StressE) during most thermal-sensitive period (specific heat stress), and temperature accumulation rate per day during grain filling (TempHRAvg) were selected to identify areas where yield formation is expected to be most prone to climate-induced stresses. Based on this selection risk maps were generated indicating whether the indicator exceeds pre-determined threshold values (RainAS < 39.4 mm, StressE >= 6 days, TempHRAvg > 14.5 °C/day). Map overlay techniques were finally used to combine the risk layers (unweighted) in order to generate a composite map indicating the overall risk potential. The overlay map contains four risk classes wherein the No risk class indicates areas where none of the selected indicators exceed the threshold value, Low risk = one indicator exceeds the threshold, Moderate risk = two indicators exceed the threshold and High risk = all indicators exceed their thresholds.

- 1. Climate change scenarios

Table S4: List of General Circulation Model (GCM) simulations downloaded from the CMIP3 archive (*Meehl et al.,* 2007) for three SRES emission scenarios (B1, A1B, A2) (Nakicenovic *et al*. 2000) for which all variables required to construct scenario data for crop modeling were available. Simulations in brackets were excluded from the ensemble. A sub-set of 11 scenarios was selected (denoted with bold X) that represents the range of uncertainty. Details on the GCMs have been summarized in IPCC (2007, Table 8.1). A larger ensemble of simulations with 24 GCMs was available from the CMIP3 archive for which not all variables required to construct scenarios for crop modeling were available.

| GCM | Institution | SRES B1 | SRES A1B | SRES  A2 |  |
| --- | --- | --- | --- | --- | --- |
| BCCR-BCM2.0 | Bjerknes Centre for Climate Research, Norway | X | X | **X** |  |
| CCCMA-CGMC3.1(T47) | Canadian Centre for Climate Modelling and Analysis | X | **X** | X |  |
| CCCMA-CGMC3.1 (T63) | Same as above | X | **X** |  |  |
| CNRM-CM3 | Météo-France | X | **X** | **X** |  |
| CSIRO-Mk3.5 | CSIRO Atmospheric Research, Australia | **X** | **X** | X |  |
| GISS-AOM | Goddard Institute for Space Studies, USA | (X) | (X) |  |  |
| GISS-EH | Same as above |  | X |  |  |
| GISS-ER | Same as above | **X** | X | X |  |
| IAP-FGOALS-g1.0 | Chinese Academy of Sciences | (X) | (X) |  |  |
| INM-CM3.0 | Institute for Numerical Mathematics, Russia | X | **X** | X |  |
| IPSL-CM4 | Institut Pierre Simon Laplace, France | X | X | **X** |  |
| MIROC3.2(hires) | Center for Climate System Research, National Institute for Enviromental Studies and Frontier Research Center for Global Change, Japan | X | X |  |  |
| MIROC3.2 (medres) | Same as above | X | **X** | X |  |
| MRI-CGCM2.3.2a | Meteorological Research Institute, Japan | X | **X** | X |  |
| Ensemble members |  | 11 (13) | 12 (14) | 9 | 32 (36) in total |

Table S5: Observed and scenario variables used for crop modeling (cf. Wolf *et al.*, 2012).

| **Observed variable (daily)** | **GCM variable (monthly mean) from which to derive deltas** | **Method of calculation** |
| --- | --- | --- |
| PR [mm] | Precipitation flux (pr) [kg m-2 s-1] | Relative change |
| TX [°C] | Mean surface temperature (tas) [K] | Absolute change |
| TN [°C] | Mean surface temperature (tas) [K] | Absolute change |
| GR [MJ m-2 d-1] | Surface downwelling shortwave flux in air (rsds) [W m-2] | Relative change |
| WS [m s-1] | Zonal (uas) and meridional wind speed (vas) [m s-1] | Absolute change in WS determined as WS = sqrt(uas*uas+vas*vas) |
| VP [hPa] | Air pressure at sea level (psl) [Pa]  Specific humidity (huss) [kg kg-1] | Absolute change in VP calculated as  VP = huss * psl / 0.62 |

**Figure S1**: Projected changes in mean temperature and precipitation during March-August relative to the baseline climate (1971-2000) presented for the time periods 2011-2040, 2041-2070 and 2071-2100 for selected locations (Turku (1), Jokioinen (2), Utti (3), Ylistaro (4), Oulu (5), Rovaniemi (6)) representing the environmental zones most relevant for agricultural production in Finland (see Fig.1). Projections are presented from six GCMs (CCCMA-CGCM3.1(T63), CSIRO-Mk3.5, GISS-ER, IPSL-CM4, MIROC3.2(medres) and BCCR-BCM2.0) runs for one particular of the following emissions scenarios B1 (low), A1B (moderate) or A2 (high).

- 1. WOFOST model simulations

*Grouping of barley cultivars*

Based on earlier work on barley cultivar diversity (e.g. Hakala *et al.*, 2012; Himanen *et al.*, 2012), for the simulations we grouped modern barley cultivars (those released after 1990 according to Plant Variety Board Official Journal (2007)) into three maturity groups, named after popular representatives: late maturing (Annabell), medium (Kustaa) and early (Kunnari). Relevant crop parameters for these cultivar groups are summarized in Table S6.

**Table S6**: Thermal requirements [^o^C day] for three modern barley cultivar groups from emergence to flowering (TSUM1), and from flowering to physiological maturity (TSUM2); assuming a common base temperature (TBASE) of 0^o^C - and indication of differences in other crop parameters.

| **Cultivars** | **TSUM1**  **[^o^C day]** | **TSUM2**  **[^o^C day]** | **TSUM (1+2)**  **[^o^C day]** | **Differences in other parameters** |
| --- | --- | --- | --- | --- |
| Annabell  Kustaa  Kunnari | 730  690  660 | 640  610  580 | 1370  1300  1240 | None  None  None |
|  |  |  |  |  |

*Crop parameter modification for different CO_2_ concentrations.*

Atmospheric CO_2_ has increased from 350 ppmv in 1985 (mid point of reference climate) to 392 ppmv in 2011 (Tubiello *et al.*, 2007; Betts *et al.*, 2011); for the next decades increases are estimated at rates between 2 and 4 ppmv per annuum (Anderson & Bows, 2008). That means, by 2025 (midpoint of 2011-2040) we may reach levels of approximately 420 to 450 ppmv; for 2055 this would be 480-570 ppmv and by 2085 this would be 540 to 690 ppmv - in line with atmospheric CO_2_ concentrations reported by IPCC (2001). We followed Rötter and Van Diepen (1994) and Rötter *et al.* (2011b) to establish crop parameters (**Table S7**) to represent shifts in crop characteristics for three higher levels of atmospheric CO_2_ concentrations (means of above ranges) as compared to the midpoint (1985) of reference climate (350 ppmv): 435 ppmv, 525 ppmv and 615 ppmv, respectively.

**Table S7**: Changes of crop parameter set (uniform for all spring barley cultivar groups) for different CO_2_ levels: Specific leaf area (SLA), maximum CO_2_ assimilation rate (AMAX occurring over indicated development stage (DVS)) and correction factor for potential evapotranspiration (CFET) under reference climate (350 ppmv) and enhanced (435, 525 and 615 ppmv) atmospheric CO_2_ concentration (with % changes in relation to current level).

| **Atmospheric CO_2_ concentration [ppmv]** | **AMAX over DVS 0-1.2**  **[kg ha^-1^ h^-1^]** | ***Change from current cv [%]*** | **CEFT**  **[-]** | ***Change from current cv [%]*** | **SLA**  **[ha leaf kg^-1^ leaf]** | ***Change from current cv [%]*** |  |
| --- | --- | --- | --- | --- | --- | --- | --- |
| 350  435  525  615 | 34.00  36.70  39.20  42.10 | *-*  *+7.9*  *+15.3*  *+23.8* | 1.00  0.98  0.95  0.91 | *-*  *-2*  *-5*  *-9* | 0.0020  0.0020  0.0020  0.0020 | *-*  *-*  *-*  *-* |  |

1. **Results**


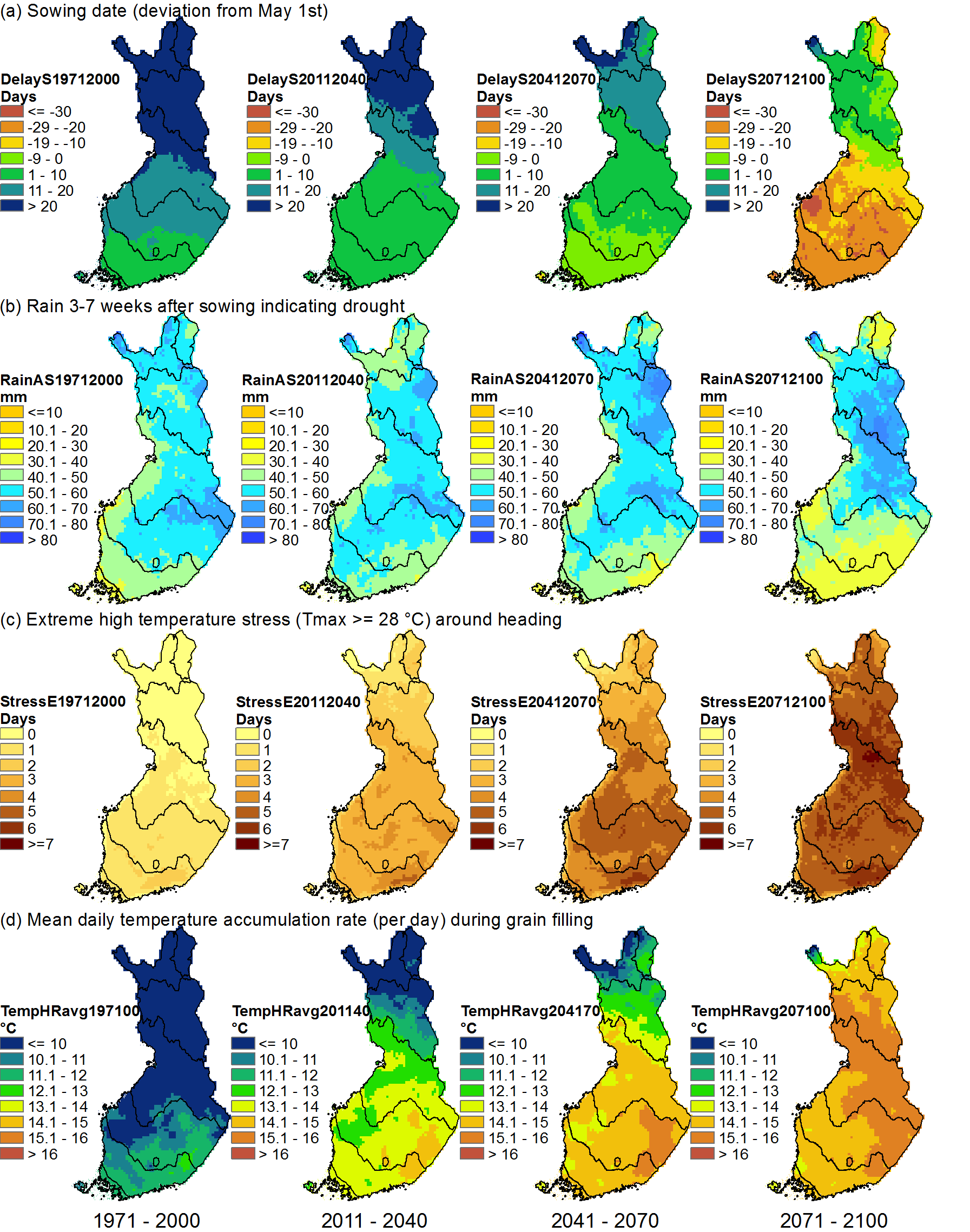


**Figure S2:** Projected changes for (a) sowing date (deviations relative to fixed date 1^st^ May) and three agroclimatic indicators: (b) early drought stress, (c) specific heat stress, and (d) yield potential reduction risk, for climate scenario 2 (warm and wet), combining SRES emissions scenario A1B with MIROC3.2(medres)(see Table S4).


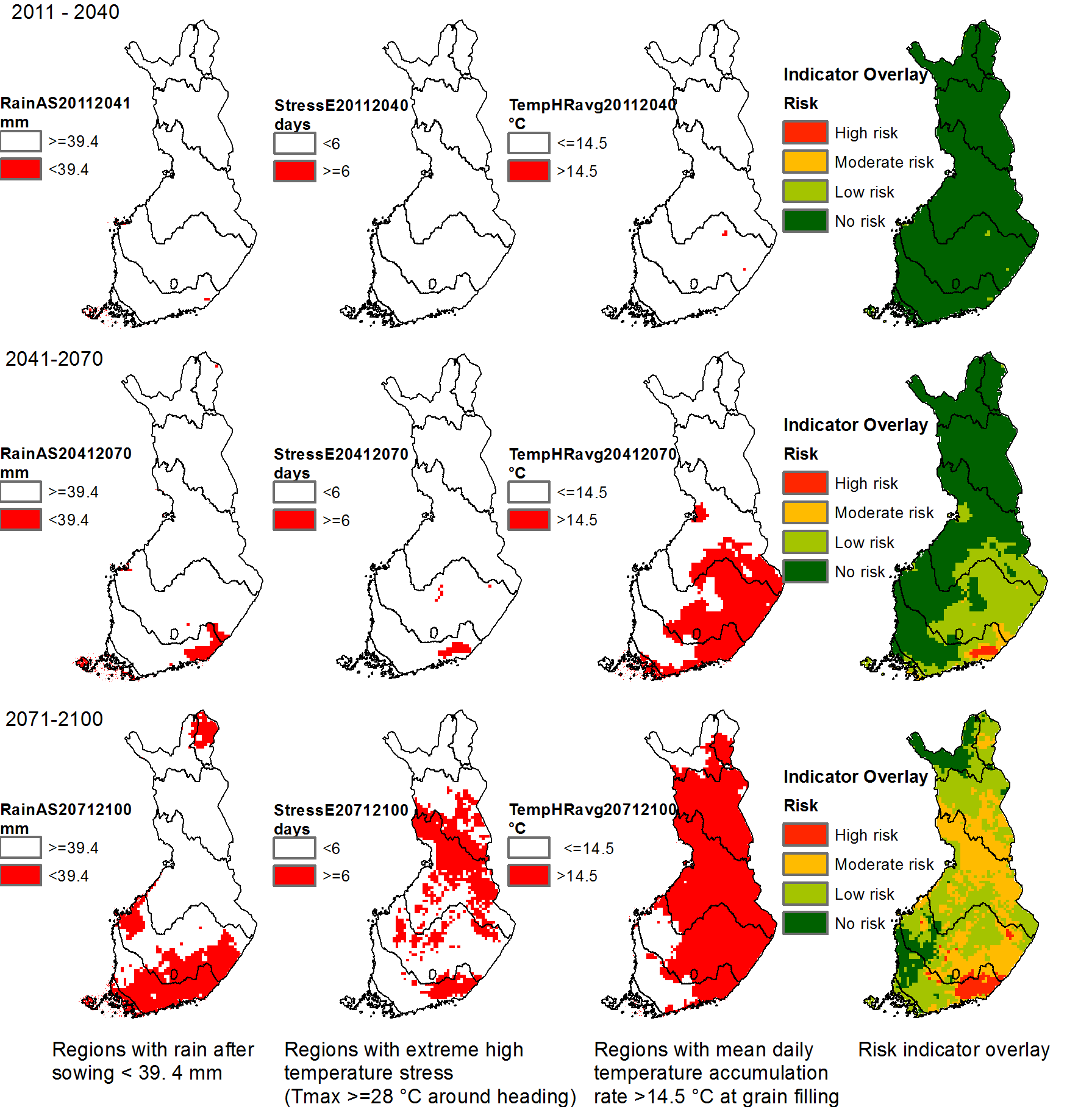


**Figure S3:** Spatial patterns of the most risk prone areas for each of these indicators using pre-determined thresholds, as well as, the overlay of all three risk factors – MIROC3.2(medres)/A1B - for each the three future time slices (2011-2040), (2041-70) and (2071-2100).

The following Figure S4 shows the secular variability of the indicator “early drought stress” for the central co-ordinates of four different grid cells, each having nearby long term weather stations (Fig. 1). Observed variability is based on historical weather records (1971-2009), and future variability simply repeats that of the baseline period (1971-2000), adjusting only the mean climate according to each of the scenarios using the delta change method (see section 2.5). Values are shown for three different climate change scenarios up to the end of the century (30 year time slices 2011-2040, 2041-2070 and 2071-2100, respectively).

**Figure S4:** Early drought stress (Rain sum 3-7 weeks after sowing) presented as 10-year moving average under current (1971-2009) and projected future climate conditions (2011-2040, 2041-2070, 2071-2100) applying delta change method and preserving the variability of the reference climate (1971-2000) for grid cells (a) Jokioinen, (b) Utti, (c) Ylistaro, (d) Oulu, representing the environmental zones most relevant for agricultural production in Finland (see Fig. 1) Climate change projections based on three GCMs x SRES combinations: GISS-ER/B1, CCCMA-CGCM3.1(T63)/A1B and IPSL-CM4/A2 (see, Table S4).

1. **Additional references for SI**

Eliasson A, Terres JM, Bamps C *et al*. (2007) *Common biophysical criteria for defining areas which are less favourable for agriculture in Europe*. Proceedings from the Expert Meeting 19–20 April 2007. The Institute for Environment and Sustainability Joint Research Centre, Ispra, Italy, 93 pp.

FAO (1979) *Yield Response to Water. Drainage and Irrigation Paper 33*. FAO, Rome, Italy.

Fischer G, van Velthuizen, Shah H, Nachtergaele FO (2002) *Global Agroecological*

*Assessment for Agriculture in 21st Century; Methodology and Results*. IIASA, Laxenburg,

Austria.

IPCC (2001) Climate change 2001: the scientific basis. *Contribution of Working Group I to the Third Assessment Report of the Intergovernmental Panel on Climate Change* (ed. by J.T. Houghton, Y. Ding, D.J. Griggs, M. Noguer, P.J. van der Linden, X. Dai, K. Maskell and C.A. Johnson), p. 881. Cambridge University Press, Cambridge.

IPCC (2007) Climate Change 2007: The Physical Science Basis. Contribution of Working Group I to the Fourth Assessment Report of the Intergovernmental Panel on Climate Change. In: Solomon S, Qin D, Manning M, Chen Z, Marquis M, Averyt KB, Tignor M, Miller HL (eds) Cambridge University Press, Cambridge, United Kingdom and New York, NY, USA, p 996

Olesen JE, Børgesen CD, Elsgaard L *et al*. (2012) Changes in time of sowing, flowing and maturity of cereals in Europe under climate change. *Food Additives and Contaminants:Part A.* DOI:10.1080/19440049.2012.712060.

Meehl GA, Covey, C, Delworth T, Latif M, McAvaney B, Mitchell JFB., Stouffer RJ, Taylor KE *et al.* (2007) The WCRP CMIP3 multi-model dataset: A new era in climate change research, *Bulletin of the American Meteorological Society,* **88**, 1383-1394.

Nakicenovic N, Alcamo J, Davis G *et al.* (2000) *Emissions Scenarios. A Special Report of Working Group III of the Intergovernmental Panel on Climate Change*. Cambridge University Press, Cambridge, UK, 599 pp.

Plant Variety Board Official Journal (2007) National list of plant varieties 2007, 2007:1 (14.5.2007), Finland.

Rötter RP, van Diepen CA (1994) *Rhine basin study: Land use projections based on biophysical and socio-economic analyses. Volume 2: Climate change impact on crop yield potentials and water use.* Report 85.2, SC-DLO and RIZA, Wageningen, The Netherlands, 152 pp.

Tubiello FN, Amthor JS, Boote KJ *et al.* (2007) Crop response to elevated CO_2_ and world food supply – A comment on “Food for Thought..” by Long *et al*., Science 312, 1918–1921, 2006. *European Journal of Agronomy,* **26**, 215–223.

Wolf J, Reidsma P, Schaap B *et al.* (2012) Assessing the adaptive capacity of agriculture in the Netherlands to the impacts of climate change under different market and policy scenarios (AgriAdaptproject). Dutch National Research Programme Climate changes Spatial Planning, Report KvR 059/12, Wageningen, The Netherlands.
